# Can Sodium Oxybate Mitigate the Symptoms of Schizophrenia?

**DOI:** 10.2174/011570159X377481250526110256

**Published:** 2025-06-04

**Authors:** Mortimer Mamelak

**Affiliations:** 1 Department of Psychiatry, Baycrest Hospital, University of Toronto, Toronto, Ontario, Canada

**Keywords:** Schizophrenia, sodium oxybate, cholinergic agonist, dopaminergic antagonist, glutamate, GABA, sleep, psychological stress, oxidative stress

## Abstract

Schizophrenia remains a therapeutic challenge. For much of its long history, the physiological basis of its symptoms and clinical presentation remained elusive. However, in recent decades, consistent anatomical and metabolic changes have been documented that can also serve as therapeutic targets. An insult to the developing nervous system in the prenatal or neonatal period appears to set the schizophrenic syndrome in motion by preventing the development of the normal circuit balance between inhibitory and excitatory neurons. In time, a reduction in the volume of frontal and temporal grey matter and a decrease in the density of dendritic spines on pyramidal neurons becomes apparent. These anatomical findings are accompanied by a reduced capacity to synthesize GABA, an increased capacity to synthesize and release dopamine, and an increased level of blood cortisol. Treatment with sodium oxybate (SO) (gammahydroxybutyrate) may make it possible to reverse these pathological features of the schizophrenic syndrome, given SO’s potential to increase neuronal levels of GABA, inhibit dopamine release and reduce blood cortisol levels. SO can also serve as a source of energy to promote the growth of the dendritic arbor on excitatory pyramidal neurons and as an antioxidant to enhance the activity of GABAergic inhibitory neurons. In this way, SO may restore the balance between the excitatory pyramidal neurons and the inhibitory GABAergic neurons in schizophrenia. In a short clinical trial, the use of SO to improve the sleep of patients with chronic schizophrenia led to a significant clinical improvement.

## 1 INTRODUCTION

More than 100 years ago, two great figures in the history of psychiatry, Emil Kraepelin and Eugen Bleuler, independently wrote that schizophrenia or dementia praecox, as it was then known, had an organic basis [1]. But that basis proved hard to identify. Organic changes were not immediately evident in the brains of patients with schizophrenia, and the disease was infamously referred to as “the graveyard of neuropathologists” [2]. Indeed, many psychiatrists were convinced that schizophrenia was a purely psychological disorder and that it did not have a somatic basis. All of this uncertainty began to change in the early 1950s with the discovery that chlorpromazine could suppress the symptoms of psychosis, delusions, and hallucinations [1]. Chlorpromazine’s therapeutic benefits were soon attributed to its actions on the dopamine (DA) receptor. In the ensuing decades, with the application of new technologies, such as positron emission tomography (PET), magnetic resonance spectroscopy (MRS), postmortem biochemical analyses and sophisticated animal models, a neuropathology for schizophrenia gradually emerged, highlighted by the reduction in the volume of the grey matter in the frontal and temporal regions of the brain without signs of gliosis. Many neurons appeared dormant but undamaged [3, 4]. An increase in DA and a decrease in GABA neurotransmission were two other key findings. Schizophrenia was recognized as a neurodevelopmental disorder that began with an insult to the developing nervous system in the prenatal or early postnatal period that prevented its normal maturation, and psychotic symptoms emerged when these developmental aberrations were exacerbated during adolescence. The current challenge is how best to manage the disease, treat its acute symptomatology, and arrest its progression.

## 2 VOLUMETRIC REDUCTION

A range of structural abnormalities has been documented in the brains of patients with schizophrenia and in those at risk for the disease [5, 6]. The most active and marked abnormalities appear to develop during the transition from prodrome to overt psychosis and to persist thereafter [7]. An increase in the volume of the pituitary gland emerges early [7-9]. Variable degrees of volumetric reduction become evident in cortical and subcortical brain regions. These volumetric reductions do not reflect neuronal degeneration as glial cell numbers are not increased [3, 5]. Volumetric reductions have been documented in the para-hippocampal gyrus, the entorhinal region, the prefrontal cortex, the hippocampus, the thalamus, the amygdala, and the globus pallidus interna [3, 10]. The most pronounced reductions appear to be in the hippocampus. In the anterior cingulate, prefrontal cortex, and hippocampal formation, there is a preferential decrease in the density of nonpyramidal GABAergic cells [3, 6]. Hippocampal abnormalities are observed in subjects at clinical high risk (CHR) for the disease and at first episode psychosis (FEP), and further shrinkage occurs over time as the illness progresses, particularly in frontal and temporal gray matter [11, 12]. In monozygotic twins, discordant for schizophrenia, volumetric reductions are more pronounced in the affected co-twin, especially in the peri-hippocampal region, and indicate that the brain abnormalities in schizophrenia are partially independent of genetic factors [13].

However, regional reductions in brain volume are not specific to schizophrenia. Reductions in hippocampal gray matter volume are common in depressive disorder, bipolar disorder, and schizophrenia spectrum disorders [14]. Volume reduction has been attributed to the dysregulation of the hypothalamic-pituitary-adrenal (HPA) axis in response to stress and to the increased production of glucocorticoids [15]. In healthy adults, the use of hydrocortisone for even 3 consecutive days can reduce hippocampal volume [16]. Prolonged stress has also been shown to reduce gray matter volume in the frontal cortex [17]. In schizophrenia, individuals with psychosis and those at CHR have been found to have elevated basal and diurnal levels of cortisol in blood and saliva, although there is considerable variability among studies [18-22].

## 3 GABAergic NEUROTRANSMISSION

A molecular defect in GABAergic neurotransmission has been a consistent finding at postmortem in the hippocampus, striatum, thalamus, and many cortical regions of the brains of schizophrenic patients [23-25]. A pronounced reduction in the expression of glutamic acid decarboxylase 67 (GAD 67) mRNA is evident in these regions. GAD67 converts glutamate to GABA in interneurons. GAD67 deficiency is accompanied by the reduced expression of GABA membrane transporter 1 (GAT1), responsible for the reuptake of released GABA into nerve terminals [24].

**Fig. (1):**
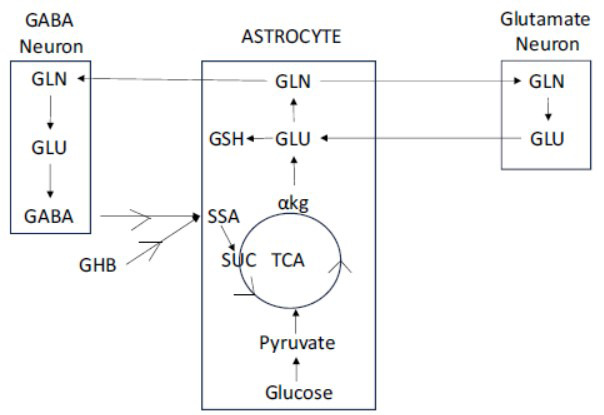
The glutamate/GABA-glutamine cycle. **Abbreviations**: αkg, alpha-ketoglutarate; GABA, gamma-aminobutyric acid; GHB, gammahydroxybutyrate; GLU, glutamate; GLN, glutamine; GSH, glutathione; SUC, succinate; SSA, succinic-semialdehyde.

In the anterior cingulate, superior temporal, dorsolateral prefrontal, and orbitofrontal cortices of individuals with schizophrenia, lower levels of GAD67 mRNA or protein have been identified specifically in GABA interneurons in layer 2 and superficial layer 3, while in the hippocampus, low levels are most prominent in sectors CA2/3 [4, 23, 26]. Studies of GAD67 mRNA expression at the cellular level show that the density of neurons with detectable levels of GAD67 mRNA is significantly decreased but that the expression level per neuron does not differ from the control value in neurons with detectable levels of GAD67 mRNA. The expression of GAD67 is driven by activity and this critically controls GABA synthesis and, thus, vesicular filling with the transmitter [27]. The rate of GABA synthesis can be reduced by blocking the synthesis of glutamine, which, in turn, reduces the production of glutamate, the precursor of GABA [28]. GABA, released into the extrasynaptic space, is taken up by astrocytes and used to synthesize glutamine and sustain neurotransmitter cycling. Reduced astrocyte GABA uptake depletes glutamine (Fig. **1**) [29, 30].

GAD67 mRNA expression is undetectable in about 50% of cortical parvalbumin (PV) interneurons [31]. These reduced levels of GAD67 mRNA are accompanied by lower levels of PV mRNA and protein. Reduced mRNA levels are also observed for somatostatin (SST), a cyclic peptide and marker for another type of cortical GABA interneuron [32, 33]. Corresponding to the reduction in GAD67, a reduction in the density of PV interneurons is one of the most reproducible findings in the postmortem brain of schizophrenic patients. In schizophrenia, pronounced deficits in SST and PV mRNA result from reduced transcript levels per neuron rather than a decrease in neuron number, arguing against neuronal death. Instead, these neurons appear to be functionally depressed and, thus, open to the possibility of full functional restoration.

As with the reductions in regional brain volumes, the deficient expression of GAD 67 transcripts is not specific to schizophrenia and has been documented at post-mortem in the brains of patients with bipolar disorder and major depressive disorder [23, 25, 34].

## 4 THE PN-PV CIRCUIT

Cortical PV interneurons innervate and are innervated by excitatory pyramidal neurons (PN) located in cortical layers 2-3 and are components of a neural circuit that mediates working memory. However, in schizophrenia, the shorter and less complex dendritic arbor, along with the reduced density of dendritic spines on PN, decreases excitatory input to PV interneurons. This leads to reduced activity in the PN-PV neural circuit and impaired working memory efficiency. Impaired working memory is a key feature of schizophrenia that can be monitored by the failure of frontal EEG power in the gamma frequency range (30-80 Hz) to increase in response to an increase in the demand for working memory [35, 36]. The reduced activity of PN reduces the need for energy, ATP, and this is reflected in the down-regulated expression of genes related to oxidative phosphorylation and energy production evident in post-mortem tissue [37, 38]. The resulting reduction in the activity of PN reduces the activity of the GABA interneurons and drives down GAD67, GAT1, PV, and SST mRNA levels [31]. Corresponding to this reduced activity, the density of glutamatergic excitatory synapses contacting PV interneurons in the prefrontal cortex is lower in schizophrenic patients than in controls [39]. The formation of these excitatory synapses on PV interneurons is regulated by Erb-B2 receptor tyrosine kinase 4 (ErbB4). Alterations of this signaling pathway in schizophrenia could reduce excitatory input to PV cells [31, 39].

What accounts for the reduced density of dendritic spines on PN? This decrease, like the reduction in the density of PV interneurons, is a very consistent finding in the postmortem brain of schizophrenic patients [40]. The apparent limitation of spine loss to smaller spines suggests that the deficit results from an inability to form or stabilize new spines [41]. Whether the reduced density of dendritic spines in schizophrenia reflects an early failure to form the normal complement of spines, a later excessive pruning of spines or some combination of the two remains uncertain [35]. In the rat, microglia transiently engulf dendritic spines on PN in the prefrontal cortex (PFC) at postnatal day 39, an age corresponding to late adolescence in humans. This phenomenon supports the hypothesis that enhanced physiological pruning by microglia during adolescence reduces the number of PFC PN dendritic spines [40, 42]. Animal studies show that stress can activate microglia and synaptic pruning [43-45]. Synapses in schizophrenia are thought to be targeted for elimination by microglia through complement-mediated pathways. Increased complement activity and an elevated microglial molecular capacity for phagocytosis are found together in the brains of patients with schizophrenia [40, 46]. PFC PNs appear to be most vulnerable to spine loss; a less pronounced decrease in spine density has been reported in the primary auditory cortex with no significant change in the visual cortex. Reduced dendrite and spine density, together with smaller pyramidal cell bodies, underlie the ventricular enlargement, reduced gray matter volume, and cortical thickness, particularly in the prefrontal and medial temporal cortices, that are consistently observed in patients with chronic schizophrenia but also at FEP. Reduced gray matter volume has even been observed in individuals at CHR for the disease. As noted earlier, these gray matter deficits may be progressive through the initial phases of the disease [40, 47].

However, a reduction in the demand or supply of energy to neurons may also reduce the density of dendritic spines. In rodents, prolonged stress or the application of corticosterone reduces the complexity of the dendritic arbor and the density of dendritic spines [48]. In response to chronic stress, fewer NMDA and AMPA receptors are delivered to dendritic spines, and glutaminergic transmission and neural activity are reduced [49]. Loss of ErbB4 localized to the dendritic spines of PN also decreases the density of dendritic spines [50]. Hypothermia, in both hibernating and non-hibernating species, reduces neural activity and the need for energy and reduces the density of dendritic spines [51]. Spine density is restored when normothermic conditions are restored and neural activity and the production of energy increase. There is a general consensus that antipsychotic drugs can alter mitochondrial function, number, and size, but it is argued that the weight of current evidence does not support attributing the metabolic depression of PN neurons to the chronic use of antipsychotic drugs [38, 52].

In contrast to the indicators of depressed metabolism in specific prefrontal cortical layers gained from studies on postmortem tissue, measurement of regional brain glucose metabolism using 18FDG-PET in the living brain of drug-free schizophrenic patients experiencing their first psychotic episode fails to reveal any differences from healthy controls in glucose utilization in either the frontal, temporal, parietal and occipital lobes, basal ganglia and thalamus [53]. Medicated patients show frontal hypometabolism compared to controls, while metabolism in drug-free patients does not differ significantly from controls. There are no differences in parietal, temporal occipital, or thalamic metabolism in schizophrenia compared to controls.

## 5 THE GLUTAMATE/GABA-GLUTAMINE CYCLE

In the brain, energy is mostly used for the neurotransmission of glutamate and GABA and specifically for glutamate/GABA-glutamine (GGG) cycling. The flow of synaptic glutamate and GABA from neurons into astrocytes and glutamine from astrocytes into neurons describes the GGG cycle (Fig. **1**) [29, 30]. An approximate 1:1 ratio exists between the rates of neuronal glucose oxidation and GGG neurotransmitter cycling [54, 55]. The flux in this cycle, which is essential for the production of glutamate and GABA, accounts for about 75% of the brain’s glucose utilization [56]. However, circumstances that interfere with the utilization of glucose disrupt GGG cycling and reduce mitochondrial oxidative phosphorylation and the production of ATP. For example, in mice exposed to chronic unpredictable mild stress, prefrontal glucose utilization declines by about 45%, together with about a 35% reduction in total ATP production [57]. Under these conditions, metabolic activity is significantly decreased in all 3 major cellular constituents of the cycle, the glutamatergic PN, the GABAergic neurons, and the astrocytes, and the production of glutamate and GABA is reduced. Gathering evidence suggests that these depressed metabolic conditions prevail in schizophrenia. Cerebrospinal fluid levels of GABA are lower in FEP, with lower levels predicting more severe symptoms [58]. PET studies reveal impaired GABA neurotransmission across multiple cortical regions in schizophrenia, particularly in anti-psychotic drug-naïve subjects [59]. Although tissue levels of glutamate and GABA measured with MRS are not consistent and include levels associated with metabolism as well as neurotransmission, a number of 7T MRS studies document reduced cortical levels of glutamate and GABA in the brain of patients with schizophrenia [60-66].

The GGG cycle is critically dependent on glycolysis and the production of NADH and pyruvate. Deficient glycolysis and the insufficient production of NADH impair the formation of ATP [56, 67]. Published data suggest that the rate of glycolysis may be suboptimal in schizophrenia. Tissue samples collected at post-mortem from the prefrontal cortex of patients with schizophrenia reveal signs of oxidative stress (OxS) and an increase in free radical formation as well as downregulation in seven out of the 10 key glycolytic enzymes. Protein levels of glyceraldehyde-3 phosphate dehydrogenase and pyruvate dehydrogenase, two enzymes prone to free radical damage, are significantly decreased [67, 68]. The significantly reduced pyruvate levels in postmortem samples of the mediodorsal thalamus of chronically treated schizophrenic patients may also reflect impaired glycolysis [69].

## 6 OXIDATIVE STRESS AND THE PARVALBUMIN INTERNEURON

7T-MRS studies in patients with schizophrenia also reveal significantly reduced levels of glutathione (GSH) in key regions of the brain. GSH is the principal molecule that maintains the redox balance in the brain [70-72]. Sydnor *et al*. [70] surveyed the findings in 255 patients, 121 with FEP, while Yang *et al*. [71] presented data garnered from 136 treatment-resistant patients with FEP. The Yang *et al.*’s meta-analysis located the reduced levels of GSH in the anterior cingulate cortex (ACC). The most recent 7T-MRS study, conducted on a cohort of 81 patients with FEP, found significantly lower levels of GSH in both the ACC and the thalamus [65]. These 7T-MRS findings suggest that OxS may be widespread in the brains of individuals with schizophrenia and are consistent with observations in chronically stressed rodents. In rats subject to restraint stress or corticosterone, brain levels of the free radical scavenging antioxidant enzymes superoxide dismutase, catalase, glutathione S-transferase, and glutathione reductase are all reduced. This reduction also applies to the major non-enzymatic antioxidant, glutathione (GSH) [73].

OxS may explain the formation of the distinct features of the PV neurons found in schizophrenia and their associated protective highly charged chondroitin sulfate proteoglycan extracellular matrix, the perineuronal net (PNN). These features, as noted earlier, consist of the reduced numbers of immunoreactive PV neurons, the reduced expression of GAD67, and the structurally abnormal PNN that are found in the hippocampus, prefrontal cortex, thalamic reticular nucleus, and other regions of the brain [74-76]. Preclinical studies reveal the sensitivity of PV neurons and their matrix to OxS [77]. Transgenic mice expressing low GSH levels (Gclm.KO) display reduced numbers of PV immunoreactive neurons in the hippocampus, prefrontal cortex, and thalamic reticular nucleus, duplicating the findings in humans. OxS precedes the development of these PV neuronal deficits and the associated defects in the PNN [74]. OxS appears to engage PV interneurons more than it does other types of interneurons, but the alterations produced by OxS can be reversed by raising the brain level of GSH with N-acetyl-cysteine (NAC).

Highly active neurons have an increased demand for GSH, which is met by a coordinated program of transcriptional regulation of antioxidant genes mediated by Ca^++^ influx through the NMDAR. NMDAR activation enhances the expression of the glutathione and thioredoxin/peroxiredoxin oxidative pathways [78]. In response, GSH reciprocally enhances the activity of the NMDAR. PV neurons are more susceptible to OxS during their postnatal maturation than they are later in life. Indeed, a transient GSH deficit during early postnatal life results in a long-term reduction of cortical PV neuron density. This vulnerability is linked to a critical period which is characterized by an immature and not yet fully formed PNN unable to protect PV neurons from OxS-induced damage [74]. Hypofunction of NMDARs during this period leads to the loss of NMDAR-dependent transcriptional support of GSH biosynthetic capacity [79, 80]. The NMDAR is sensitive to the redox environment, and its activity is reduced under oxidizing conditions. Anomalous development of PV neurons follows in the wake of GSH depletion. In rodents, even at maturity, reduced activity of the NMDAR and the loss of neuronal PV and GAD67 expression come about when the stress of social isolation or exposure to ketamine activates NADPH oxidase, leading to superoxide production. This oxidative stress, in turn, oxidizes and impairs NMDAR function [81-83].

Neurons receive significant antioxidant support from surrounding astrocytes [79]. The mechanism of protection involves the increased synthesis of astrocytic GSH, which is released, broken down, and taken up by neurons to be used as precursors for their own GSH synthesis. Astrocytes contain one of the highest cytosolic concentrations of GSH amongst mammalian cells. GSH biosynthetic enzymes, γ-glutamate cysteine ligase (GCL) and GSH synthase are highly expressed in astrocytes and together generate GSH from its immediate amino acid precursors, glutamate, cysteine, and glycine [84]. Expression of the gene for γ-glutamate cysteine ligase modifier, a subunit of GCL, is decreased in schizophrenia [85].

## 7 AN ANIMAL MODEL OF SCHIZOPHRENIA

An animal model of schizophrenia has been created by administering methylazoxymethanol acetate (MAM) to pregnant rats at gestational day 17, corresponding to the second trimester in humans when pathological events are most likely to lead to the later development of schizophrenia [86]. MAM is a mitogen that induces aberrant DNA methylation and abnormal neurogenesis. It also conjugates with and depletes cerebral glutathione (GSH), thereby reducing the cell’s antioxidant capacity and increasing vulnerability to OxS [87, 88]. MAM initiates a process of pathological neurodevelopment with behavioural and neuroanatomical changes that are not fully evident until the rat reaches puberty. For example, MAM-treated rats initially show increases in locomotor activity following amphetamine administration comparable to control rats [89, 90]. However, similar to schizophrenia, enhanced sensitivity to psychomotor stimulants only occurs after puberty when an increase in dopamine neuron population activity is present. The increase in spontaneously active mesolimbic dopamine neurons follows from the progressive decrease in PV-expressing interneurons in the hippocampus produced by the gestational administration of MAM [89, 91].

Peripubertal administration of diazepam to MAM-treated rats attenuates the decrease in PV expression and prevents the emergence of the hyperdopaminergic state in adult rats [92]. This finding suggests that stress relief in patients at CHR may prevent the development of acute psychosis. Agents that raise the brain level of the antioxidant GSH may also abort this pathological process. Thus, perinatal treatment of rats with ketamine induces a long-lasting increase in OxS reflected in a reduced GSH/GSSH ratio, increased superoxide production, and reduced neuronal PV expression. These changes can be mitigated by the administration of NAC, the antioxidant and precursor of GSH [93]. Comparable therapeutic benefits have also been documented in the rat neonatal ventral hippocampal lesion model with the use of NAC to prevent the falloff in PV interneuron activity and other behavioural anomalies which emerge in the adult [94].

## 8 DOPAMINE NEUROTRANSMISSION IN SCHIZOPHRENIA

Technical studies employing PET and single photon emission computed tomography (SPECT) have revealed that the capacity for striatal DA synthesis and release is increased in schizophrenia without any major change in DA D2/3 receptor availability and that this increased capacity correlates with the severity of the psychosis. The dopaminergic function is significantly greater in schizophrenic patients than it is in healthy control subjects, particularly in the associative striatum and, to a lesser, although still significant extent, in the sensorimotor striatum but not in the limbic region. Fluorodopa uptake is increased, and amphetamine-induced DA release is abnormally high in the associative striatum, consistent with the increase in DA neuron responsiveness and the excessive DA release that elevates striatal levels of synaptic DA [95-97].

Presynaptic DA function in individuals at CHR for psychosis is also most abnormal in the associative striatum, and the conversion of CHR to psychosis can be plotted to a progressive increase in DA synthesis capacity in this region [98].

Most dopaminergic neuron cell bodies are located in the midbrain, particularly in the ventral tegmental area and the more lateral substantia nigra. They have dense projections to the striatum but also project to the frontal cortex and several other brain regions [97]. In rodents, the medial DA neurons of the ventral tegmental area (VTA) innervate the reward-related ventral striatal regions, including the nucleus accumbens. More laterally located DA neurons project to the associative striatum, and increased presynaptic DA activity in these neurons correlates best with the emergence of psychosis. The DA neurons of the substantia nigra project to the dorsal striatum and mediate motor functions [99].

DA neurons, *in vivo*, are either hyperpolarized and inactive or display an irregular, 4 to 10 Hz, single-spike firing pattern [86, 100]. They can also fire in phasic bursts that lead to a much greater release of DA [101]. Distinct afferent pathways to the VTA determine the firing pattern of DA neurons and, thus, the level of DA in the striatum. Under resting conditions, about half of all DA neurons in the VTA are hyperpolarized and not spontaneously active. One commonly described pathway that mediates the activation of DA neurons in schizophrenia originates in the hippocampus, where metabolically depressed GABAergic PV interneurons fail to inhibit the hippocampal glutamatergic pyramidal neurons that project to the nucleus accumbens. This, in turn, activates GABAergic interneurons in the nucleus accumbens that inhibit the GABAergic interneurons in the ventral pallidum, which normally hyperpolarize and inhibit the activity of DA VTA dopaminergic neurons. Activation of the hippocampal-nucleus accumbens-ventral pallidum circuit disinhibits these neurons and increases the population of active DA VTA neurons [102]. The activation of this circuit may account for the hippocampal hyperactivity consistently observed in patients with chronic schizophrenia and in high-risk individuals who progress to psychosis, as well as for the raised striatal glutamate levels in subjects with the prodromal symptoms of schizophrenia and in those experiencing their FEP [103-106].

Metabolically depressed cortical GABA interneurons may similarly fail to inhibit cortical glutamatergic neurons that project to the basal ganglia, which then trigger the disinhibition of striatal dopamine neurons [97]. Depressed infralimbic prefrontal activity may also increase DA neuronal activity through a complex circuit that engages the thalamus. As rat models reveal, reduced corticothalamic activity deactivates the midline thalamic reticular nucleus (TRN), which projects to the thalamic nucleus reunions (RE) and normally inhibits it. Disinhibition of the RE triggers the activity of RE neurons that activate glutamatergic hippocampal pyramidal neurons and the hippocampal-nucleus accumbens-ventral pallidum circuit and results in an increase in the number of active DA neurons [107, 108]. A significant reduction in the number of functional PV interneurons in the TRN in schizophrenia likely further augments the activity of RE neurons and the baseline population of active DA neurons [109, 110].

The majority of antipsychotic drugs act by blocking postsynaptic D2- DA receptors. This results in the feedback activation of the DA system that produces a depolarization block of DA neurons and reduces the population of active DA neurons and their hyper-responsiveness to stimuli [111, 112]. D2-DA partial agonists, such as aripiprazole, do not induce a depolarization block but instead downregulate DA neuron activity by acting as agonists on presynaptic D2-DA receptors [113].

The clinical benefits of antipsychotic drugs acting on D2 receptors stem mainly from their ability to reduce the positive symptoms of schizophrenia, *i.e*., the hallucinations, delusions, and disordered thinking, but they do not improve the negative symptoms of the disease, the impaired attention, concentration, memory, and executive functions. It has long been known that arecoline, an alkaloid and muscarinic cholinergic receptor (mAChR) agonist found in betel nuts, can improve the symptoms of schizophrenia [114, 115]. More recently, it has been shown that xanomeline, a synthetic mAChR agonist derived from arecoline, can alleviate the positive symptoms of schizophrenia and improve the negative symptoms of the disease without producing the common side effects of the antipsychotic D2 receptor antagonists, weight gain, extrapyramidal effects, and sedation. Xanomeline has been shown to improve positive and negative symptoms of schizophrenia after as little time as one week [114-116].

## 9 CHOLINERGIC AGONISTS AND DOPAMINE NEUROTRANSMISSION

mAChR agonists act at sites in the striatum and midbrain to reduce the activity of VTA DA neurons. In the striatum, mAChR is expressed on cholinergic interneurons in the nucleus accumbens as well as on medium spiny neurons. Activation of the mAChR on the cholinergic interneurons reduces acetylcholine release and blocks the subsequent stimulation of DA release by the actions of acetylcholine on the nicotinic cholinergic receptors (nAChR) on DA terminals [114, 115]. Inhibition of local DA release may also occur by activation of mAChR co-expressed with D1 receptors on the medium spiny neurons. These neurons contain GABA as the major neurotransmitter and give rise to the so-called striatonigral pathway. Activation of mAChR on these GABAergic striatal projection neurons inhibits the release of GABA, and this, in turn, increases the activity of inhibitory GABAergic interneurons which act on VTA DA neurons. D1-mAChR-KO mice show a significant increase in basal DA efflux in the nucleus accumbens [117]. There is also evidence that the activation of mAChR on D1 receptor-spiny projection neurons increases the release of the endocannabinoid 2-arachidonoylglycerol and that this, in turn, activates cannabinoid CB2 receptors on presynaptic terminals of DA to produce a sustained inhibition of DA release [118, 119].

There are 5 mAChR subtypes, M1, M2, M3, M4 and M5 [114, 115]. Activation of the M1, M3, and M5 receptor subtypes facilitates neuronal excitation, while the M2 and M4 subtypes are inhibitory. The M1 receptor is largely expressed postsynaptically, particularly in the cerebral cortex and hippocampus. The M2 receptor primarily functions as a presynaptic inhibitory autoreceptor on acetylcholine-producing neurons or as an inhibitory heteroreceptor on neurons that release glutamate, GABA, and DA. The M2 receptor is highly expressed in the brainstem and thalamus and is expressed at low levels in cortical regions. Xanomeline has only modest selectivity across these five mAChRs but prefers the M1/M4 receptors.

In the midbrain, stimulation of M2 and M4 mAChRs present on the soma/dendrites of the cholinergic and noncholinergic membranes of lateral dorsal tegmental (LDT) and pedunculopontine (PPT) neurons activates GIRK channels that hyperpolarize the neuronal membrane and inhibit the release of neurotransmitters [120]. These midbrain LDT and PPT excitatory projections contain both cholinergic and glutamatergic neurons and regulate the firing and bursting activity of VTA DA neurons [100]. GABAB receptors are also located on cholinergic LDT neurons. Stimulation of these receptors by GABAB agonists also hyperpolarizes the neuronal membrane and does so by activating the same GIRK channels as cholinergic agonists [121]. The cholinergic agonist carbachol and the GABAB agonist baclofen both inhibit LDT activity and reduce the firing of VTA DA neurons [100].

Stress increases the excitability of LDT projections. The effects of stress are mediated by the release of corticotropin-releasing hormone (CRH), which binds to the receptor CRHR1 on LDT cholinergic neurons and markedly increases their firing rate. The release of acetylcholine increases VTA DA firing by direct activation of the nAChR on DA neurons, but acetylcholine also augments the release of glutamate by activating presynaptic nAChR, which then stimulates the NMDA receptors on VTA DA neurons. More than 90% of cholinergic neurons in the LDT express CRHR1 receptors. Locally antagonizing CRHR1 in the LDT will reverse the activation of cholinergic neurons and prevent stress-induced changes in VTA DA activity [122]. Cortisol, released during stress, increases DA neuron activity and DA release by acting on glucocorticoid receptors located on dopaminoceptive neurons in the nucleus accumbens, which, in turn, feedback to activate DA neurons in the VTA [123].

## 10 SODIUM OXYBATE (GAMMAHYDROXYBUTYRATE)

SO, a known GABAB agonist, also acts as a cholinergic agonist, and it may, therefore, have a unique place in the treatment of schizophrenia. Much like baclofen and carbachol, SO hyperpolarizes immunohistochemically identified cholinergic LDT neurons by activating the GIRK channels on their neuronal membranes [121, 124]. Much like carbachol and baclofen, it should reduce the activity of VTA DA neurons and the release of DA.

In addition, SO can also block DA neurotransmission by acting directly on DA neurons to reduce the striatal release of DA. SO has been shown to produce a concentration-dependent membrane hyperpolarization of DA neurons in the rat VTA by its actions on GABAB receptors [125]. These receptors are also coupled to GIRK channels, and the efficiency of GABAB receptor-GIRK channel coupling is increased by repetitive exposure to SO [126]. Experimental work conducted on rat substantia nigra dopaminergic neurons demonstrate that intravenous administration of SO or baclofen induces a dose-dependent decrease in the firing rate of nigral DA neurons, a dose-dependent regularisation of the firing pattern, and a significant reduction in burst firing that can all be blocked by a GABAB antagonist [127]. Studies using microdialysis reveal that the systemic administration of SO reduces striatal DA release. This has been held to account for the rise in striatal levels of DA after the systemic administration of SO [128-130]. The reduction of striatal dopamine release by SO likely also accounts for its marked inhibition of d-amphetamine-induced locomotor activity [131]. D-amphetamine-induced hyperlocomotion is used as a rat model of acute psychosis [132].

PET scans have been used to show that SO reduces striatal DA release in the brain by measuring the competition between DA and [C-11] raclopride, a D2 receptor radioligand and antagonist for the D2 receptor. High doses of SO, 400 mg/kg i.p. of the prodrug gamma-butyrolactone in the mouse, and 200 mg/kg i.v. or 400 mg/kg i.v. in the baboon, increase raclopride binding and reflect the reduced levels of synaptic DA [133, 134]. However, in humans, a low oral dose of 3 grams of SO failed to significantly increase raclopride binding, except possibly in the limbic striatum [135].

The therapeutic benefits of dopaminergic antagonists and cholinergic agonists in schizophrenia raise the possibility that SO may further enhance the clinical effectiveness of these drugs. SO has the pharmacological properties of both a dopaminergic antagonist and a cholinergic agonist. SO may even have an independent place in the treatment of schizophrenia.

## 11 SODIUM OXYBATE AND SLEEP

In addition to activating VTA DA neurons, LDT cholinergic neurons promote wakefulness and REM sleep with projections to the thalamus and brain stem nuclei [120]. Extracellular acetylcholine concentrations in the thalamus are at their highest levels during wakefulness and REM sleep and significantly lower during slow-wave sleep [136]. Corresponding to this, the frequency at which mesopontine cholinergic neurons discharge is lowest during slow-wave sleep [137]. As acetylcholine levels decline, GABAergic thalamic reticular neurons escape from inhibition, and bursts of action potentials are generated in reticular nucleus neurons due to activation of low-threshold (T-type) calcium channels. The release of GABA stimulates post-synaptic GABAB receptors and progressively hyperpolarizes thalamocortical neurons by opening GIRK channels. Initial levels of thalamocortical hyperpolarization are sufficient to generate sleep spindles, which are relayed to the cerebral cortex, but as the thalamocortical membrane becomes even more hyperpolarized, slow wave rhythms at 0.5 Hz- 4 Hz supervene [138]. These naturally occurring slow wave rhythms can be blocked by GABAB antagonists [139]. A similar progressive hyperpolarization of thalamocortical neurons and sequential induction of spindles and slow waves can be induced in rats and cats with increasing doses of SO and blocked with GABAB receptor antagonists [140].

In schizophrenia, a deficit of sleep spindles and slow waves has been documented in drug-free individuals as early as the first psychotic break [141]. First-degree relatives of schizophrenic patients may also have reduced spindle activity. The reduction in spindle density is localized to the frontal region, where a significant reduction in slow wave activity during sleep may also be apparent. Poor sleep is a common complaint in patients suffering from schizophrenia and a pervasive complaint in those at CHR for the disease in whom it is linked to greater positive and negative symptoms and worse overall functioning. Impaired sleep may herald the onset of the first psychotic episode or the decompensation of chronic patients in remission [141-145]. Sleep problems are highly prevalent in the first psychotic episode and are associated with suicidal ideation and high levels of psychopathology [146]. A 4-week trial of SO in 8 schizophrenic patients found that the nocturnal application of SO prolonged stage 2 (spindling) sleep and significantly increased slow-wave sleep. Measures of sleep quality and daytime somnolence were significantly improved, and this was true as well for the total score on the Schizophrenia Positive and Negative Symptom Scale (PANSS) and for the score on the negative symptoms of this scale [147].

## 12 SODIUM OXYBATE AND STRESS

While the use of SO at night to improve the sleep of individuals at CHR for schizophrenia may improve their functional ability, its early application may delay or even prevent a psychotic break. A prospective population-based study of adolescents and young adults found that the association between severe recent life stress and the risk of psychosis was strongest among individuals with a history of childhood adversity [148]. Childhood trauma appears to sensitize the neural mechanisms mediating stress and, specifically, the reactivity of the hypothalamic-pituitary axis (HPA) and the VTA DA neurons [6].

Although the neural mechanisms underlying the sensitization to stress are unknown, research on rodents has shown that exposure to severe or persistent stress/trauma can augment the DA response to subsequent stressors. In young ‘adolescent’ rats, restraint stress plus daily foot shock increases the number of spontaneously active DA neurons in the VTA in the adult rat as well as the amphetamine-induced locomotor response. Identical stress administered to the adult rat fails to achieve these ends. The increased DA reactivity that develops in the stressed rats duplicates the increased reactivity found in schizophrenic patients [95, 149]. Severe stress early in life has been proposed to alter the set-point of the HPA axis. The increase in DA reactivity has been attributed to the increased release of CRH and cortisol. As noted earlier, although there is considerable heterogenicity among studies, individuals with psychosis and those at CHR have higher basal and diurnal cortisol levels in blood and saliva than healthy control subjects but diminished salivary cortisol secretion in response to acute stress exposure and awakening [18, 19]. Cortisol levels correlate with symptom severity and are higher in those who become psychotic compared to healthy control subjects and CHR subjects who remit [150]. Although not consistently elevated, blood cortisol levels are found in individuals with FEP [20-22].

SO can be used to reduce blood levels of cortisol and block the peripheral effects of CRH. Cortisol levels begin to rise during the early morning hours and peak during the first hour after awakening. A single dose of SO during those early morning hours significantly attenuates the cortisol awakening response in healthy humans [151]. A single dose of SO lasts about 3 hours. Given repeatedly during the day, SO reduces the hypercortisolism of the alcohol withdrawal syndrome more effectively than diazepam [152]. By acting on the GABAB receptors on LDT cholinergic neurons, SO may antagonize the activation of these neurons by CRH and the consequent stimulation of DA release.

Could the early application of SO in patients at HCR for schizophrenia contain the increased release of DA, suppress cortisol release, attenuate the dendritic atrophy of the PN, and impede the progressive atrophy of the cerebral gray matter? These phenomena have all been attributed to the activation of the HPA axis.

## 13 PARVALBUMIN-PYRAMIDAL NEURON CIRCUIT REPAIR

The loss of balance between the PV inhibitory neurons and excitatory PN described earlier is a key pathological feature of the brain in schizophrenia [35]. This imbalance is established early in life, in the prenatal and perinatal period, in response to genetic predisposition and to a host of environmental factors. Among the latter are prenatal maternal stress, maternal infection, and immune activation, obstetrical complications such as low birth weight, prematurity, and hypoxia, and early life stress [153, 154]. Once established, the PV-PN circuit imbalance persists in time and may be further aggravated by stress in later years [95, 149]. PV-PN circuit imbalance impacts social behaviour, fear processing, working memory, and attention. It critically alters the control of DA release [74]. Can this imbalance be corrected? Specifically, can the reduced activity of the PV neurons throughout the brain be reversed and the production of GABA restored to normal levels? Can the excitability and energy production of PN be restored to normal levels?

Glutamate and GABA are both taken up by astrocytes following their discharge into the synaptic space (Fig. **1**). In the astrocytes, about 75% of the glutamate is converted to glutamine and reenters the GGG cycle, while the remaining 25% is oxidatively degraded, although a fraction is utilized to synthesize GSH [84, 155]. GABA generates succinate after it is taken up following two sequential enzymatic reactions, GABA transaminase and succinic semialdehyde dehydrogenase. Succinate, in turn, is converted to α-ketoglutarate along the TCA [156]. Thus, astrocytes can also use GABA to support glutamine synthesis and sustain neurotransmitter recycling. Inhibition of astrocyte GABA uptake depletes glutamine [29, 30].

Like GABA, SO is metabolized to succinic semialdehyde and then to succinate, and thus, it, too, should be able to promote GGG cycling and the synthesis of GSH [156] (Fig. **1**). In the course of its metabolism through the TCA, malate dehydrogenase and isocitrate dehydrogenase generate NADPH, and thus, SO creates a powerful antioxidative cellular environment [157]. At the same time, the metabolism of SO and succinate generates ATP energy. Indeed, SO can replace glucose as a fuel for the brain [158].

By promoting optimal levels of flux and glutamate synthesis through the TCA cycle, SO may be able to able to maintain adequate levels of glutamine for transfer to glutamatergic and GABAergic neurons for the synthesis of their specific neurotransmitters. Glutamate and GABA both appear to be depleted in the crucial cortical level 3 PV-PN circuit [159]. Maintaining adequate supplies of glutamate and GABA would be particularly helpful under conditions of stress when, as demonstrated in mice, increased demand reduces glutamatergic and GABAergic neuronal metabolism and neurotransmitter cycling [57, 160]. In rodents and humans, experimental evidence indicates that SO can increase glutamate production. Studies using microdialysis in freely moving rats reveal that low doses of SO infused into the hippocampus increase extracellular levels of glutamate. In-vitro studies demonstrate that glutamate efflux is increased in hippocampal synaptosomes exposed to SO [161]. In normal human subjects, studies using magnetic resonance spectroscopy show that a single nocturnal dose of SO significantly increases the morning glutamate signal in the anterior cingulate cortex and improves vigilance [162].

In addition to promoting glutamate neurotransmission, SO may further increase the activity of PN by augmenting neuronal succinate levels and energy production along the TCA. Increased energy production, in turn, could stimulate the growth of the dendritic tree. The ability of SO to block the effects of cortisol would further stimulate this growth and enhance the excitability and activity of PN.

A “two-hit” neurodevelopmental model for schizophrenia has been proposed to account for the excessive pruning of the dendritic tree [163, 164]. According to this model, perinatal immune activation primes the microglia, which then becomes excessively activated in response to stress later in life. This leads to the abnormal synaptic pruning that creates the cortical pathology and characteristic features of schizophrenia. Agonists of GABAB receptors prevent the activation of microglia by inducing an increase in K^+^ conductance that hyperpolarizes the microglial membrane [165]. SO is a GABAB agonist with a known capacity to increase K^+^ conductance and hyperpolarize cell membranes [124, 166].

Furthermore, SO may inhibit microglial activation by acting epigenetically. SO is a known inhibitor of the class 1 histone deacetylase, histone deacetylase 3 (HDAC3), the most widely expressed class 1 HDAC in the brain. HDAC3 inhibitors have been shown to act epigenetically to inhibit microglial activation [167-169]. In its capacity as a class 1 histone deacetylase inhibitor, SO may also act epigenetically to increase the expression of GAD67 and the synthesis of GABA in PV interneurons, further increasing neurotransmitter levels and cycling through the GGG [170].

Finally, SO may be able to increase the activity of GABAergic interneurons by preventing the oxidation and hypofunction of the NMDAR. Oxidation and hypofunction of the NMDAR, as induced in animal models by the activation of NADPH oxidase and the generation of oxidative stress, reduces neuronal activity, GAD67 levels, PV levels, and GABA production [81-83]. Apocynin, an inhibitor of NADPH oxidase, prevents these effects. SO, with its capacity to generate NADPH, inhibit NADPH oxidase, and promote the formation of glutathione, may also prevent the oxidation of the NMDAR and maintain optimal levels of PV GABA interneuron activity [157].

In short, through a variety of mechanisms, SO may be able to improve the function and activity of the PV-PN circuit in the brain of patients with schizophrenia.

## CONCLUSION

Can treatment with SO mitigate the symptoms of schizophrenia and alter the course of the disease? The results of a brief 4-week trial of SO in patients with chronic schizophrenia are encouraging. SO improved sleep at night and alertness during the day and it significantly alleviated the clinical symptoms of the disease [147]. Would the continued use of SO have led to an even greater improvement over time? This question is particularly relevant to patients at CHR for schizophrenia. Would the sustained use of SO improve overall function and act prophylactically to delay and even prevent the onset of psychosis? The short 30 to 60-minute half-life of SO makes it possible to apply SO repeatedly at night and again during the day [152]. Given its capacity to contain the release of dopamine, block the actions of cortisol, and prevent the depletion of glutamate and GABA under conditions of high demand, could SO be used to rapidly terminate an acute psychotic event? The duration of untreated psychosis is a critical prognostic factor, and clinical observations suggest that an active psychosis can be progressively detrimental until treated [99].

SO’s place in the management of schizophrenia remains to be determined.

## AUTHORS' CONTRIBUTIONS

The author confirms sole responsibility for the following: study conception and design, data collection, analysis and interpretation of results, and manuscript preparation.

## LIST OF ABBREVIATIONS

ACC: Anterior Cingulate Cortex
CHR: Clinical High Risk
CHR: Corticotropin-releasing Hormone
DA: Dopamine
FEP: First Episode Psychosis
GGG: Glutamate/GABA-glutamine
HPA: Hypothalamic-pituitary Axis
LTD: Lateral Dorsal Tegmental
MAM: Methylazoxymethanol Acetate
MRS: Magnetic Resonance Spectroscopy
OxS: Oxidative Stress
PET: Positron Emission Tomography
PFC: Prefrontal Cortex
PN: Pyramidal Neurons
PPT: Pedunculopontine
PV: Parvalbumin
SO: Sodium Oxybate
SST: Somatostatin
TRN: Thalamic Reticular Nucleus
VTA: Ventral Tegmental Area
